# A monoclonal antibody-based indirect competitive enzyme-linked immunosorbent assay for flubendiamide detection

**DOI:** 10.1038/s41598-019-38649-w

**Published:** 2019-02-14

**Authors:** Qibo Li, Yongliang Cui, Min Liao, Tong Feng, Guiyu Tan, Baomin Wang, Shangzhong Liu

**Affiliations:** 1 College of Science, China Agricultural University, Beijing, 100193 China; 2 Citrus Research Institute, Southwest University, Chongqing, 400712 China; 3 College of Agronomy and Biotechnology, China Agricultural University, Beijing, 100193 China

## Abstract

Flubendiamide (FD), the first commercial phthalic acid diamide that targets insect ryanodine receptor (RyRs), has played an important role in pest management. With its extensive worldwide application, a rapid and convenient method to detect its existence in the environment is necessary. In this study, an indirect competitive enzyme-linked immunosorbent assay (icELISA) was developed to analyse FD residue on environmental and food samples. The established icELISA showed a half maximal inhibition concentration (IC_50_) of 17.25 µg L^−1^, with a working range of 4.06–103.59 µg L^−1^ for FD, and showed no cross-reactivity with chlorantraniliprole, cyantraniliprole, and several FD analogues. Average FD recoveries from spinach, tap water, and soil samples were 89.3–112.3%, 93.0–102.1%, and 86.9–97.6%, respectively. Meanwhile, FD detection results of icELISA were compared with those of ultra-high-performance liquid chromatography-tandem mass spectrometry (UPLC-MS/MS). The comparable results verified that icELISA was suitable for rapid detection of FD residue in environmental and agricultural samples.

## Introduction

Flubendiamide (N^2^-[1,1-dimethyl-2-(methylsulphonyl)ethyl]-3-iodo-N^1^-[2-methyl-4-[1,2,2,2-tetrafluoro-1-(trifluoromethyl)ethyl]phenyl]-1,2-benzenedicarboxamide) is a novel class of insecticide with a unique chemical structure that has garnered interest worldwide. Developed by Bayer Crop Science and Nihon Nohyaku in 2007, it became the first commercialised phthalic diamide insecticide that targets insect RyRs. It is characterised by its broad insecticidal spectra, extremely high intrinsic potency, remarkable selectivity, low ecotoxicity, and low residue levels^1–3^. At present, FD is used to control lepidopterous insects, including *Helicoverpa* spp., *Heliothis* spp., *Spodoptera* spp., *Plutella* spp., *Trichoplusia* spp., and *Hyrotis* spp., as part of pesticide resistance management and integrated pest management programs^4^. Therefore, it is applied extensively on crops, vegetables, and fruits^5^.

According to a recent report by the United States Environmental Protection Agency (US EPA), the continued use of FD will result in unreasonable adverse effects on the environment due to one degradation product of FD in water is highly toxic to fish. The FD registrations of over 200 crops in the US and rice in China were cancelled in 2016, its products still ranked in the top ten of the global pesticide market^6^. In particular, due to its excellent performance in pest control, FD is still used worldwide against bollworm outbreak. However, with an extensive use and possible misuse of FD, environmental risks and food safety due to its residue become an important concern. Therefore, to effectively monitor FD residue and maintain food and environmental safety, it is important to develop a quick and convenient FD detection method.

Many analytical methods have been developed for FD residue detection in environmental and food samples, including high-performance liquid chromatography (HPLC) with UV-visible detection (UVD)^7–10^, HPLC with photodiode array detection (DAD)^11,12^, and tandem mass spectrometry (MS/MS)^13^. Conventional instrumental methods exerted remarkable sensitivity, selectivity, accuracy, and reliability, but usually require expensive instruments, large consumption of solvents, highly skilled professionals, and time-consuming sample preparation.

The immunology-based enzyme-linked immunosorbent assay (ELISA) has been extensively applied in the detection of environmental contaminants. Compared to conventional instrumental methods, ELISA is a rapid and sensitive method which requires only small quantities of test materials and simple pre-treatment of sample^14–16^. The indirect competitive enzyme-linked immunosorbent assay (icELISA) is based on the competition between the immobilised antigen and an unknown amount of analyte to bind with a small fixed amount of antibody^17^. The antibody bound to the immobilised antigen would also bind to a specific enzyme-labelled second antibody. The colour intensity obtained is inversely proportional to the amount of analyte being investigated^18^. Such immunoassay is suitable for monitoring trace amounts of pesticides in a large number of environmental and food samples.

To our knowledge, there is no report of monoclonal antibody (mAb)-based ELISA to monitor residual FD in the environment and food samples. In this study, a hapten was designed and synthesised based on FD structural characteristics, and anti-FD monoclonal antibody (mAb) and icELISA for the analysis of FD were developed. Through verification by analytical method based on UPLC-MS/MS, the developed icELISA is a confirmed sensitive and effective method to quantitatively determine trace FD in the environment and agricultural samples.

## Results and Discussion

### Preparation of FD haptens and development of mAb

**Figure 1:**
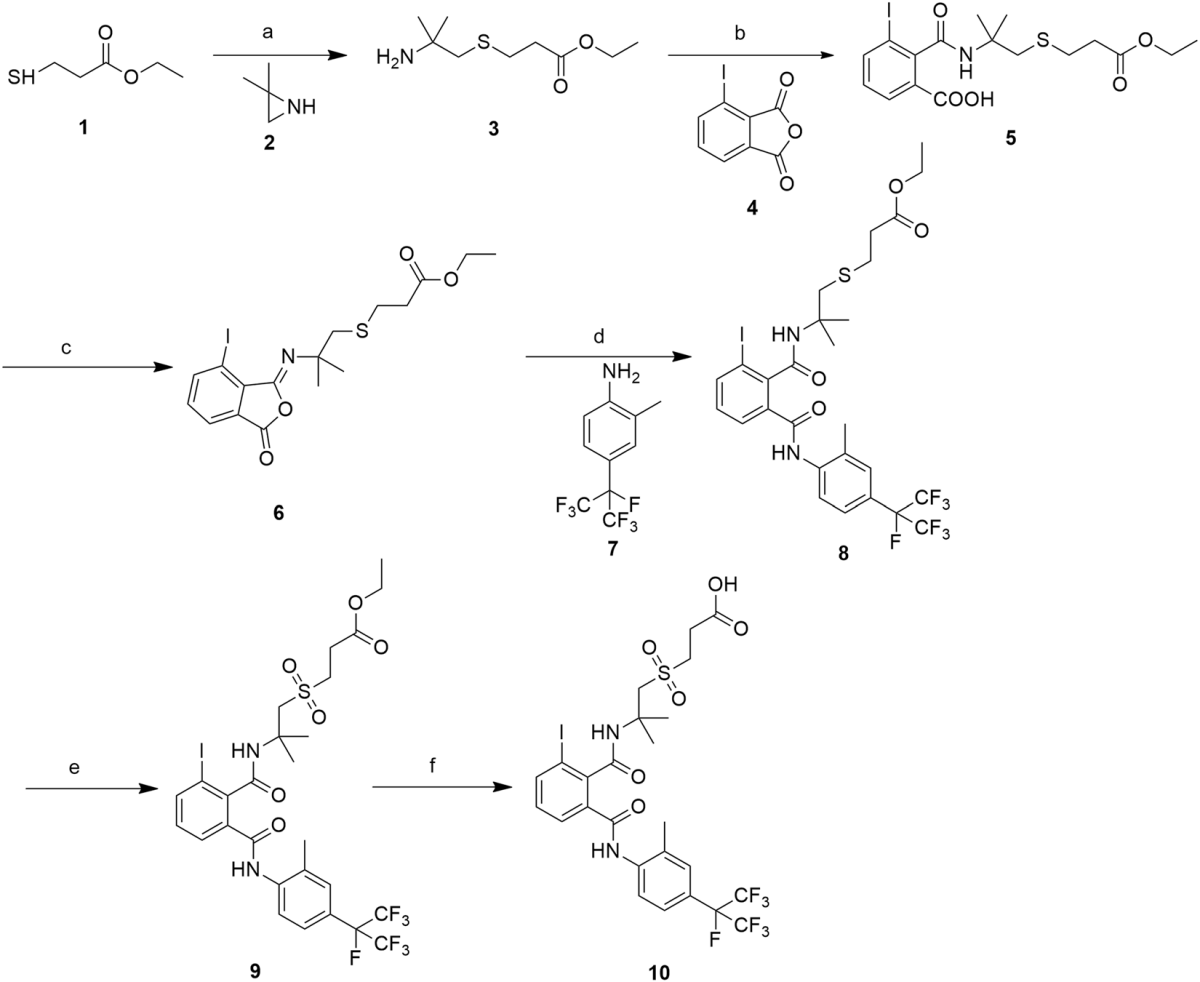
Route of FD hapten synthesis. Reagents and conditions: (**a**) 1,2-Dimethoxyethane, 85 °C, 3 h; (**b**) Et_3_N, CH_2_Cl_2_, 25 °C, 5 h, HCl; (**c**) (CF_3_CO)_2_O, toluene, 25 °C, 2 h; (**d**) CF_3_COOH, CH_3_CN, 25 °C, 2 h; (**e**) m-CPBA, CH_2_Cl_2_, 0-25 °C, 2 h; (**f**) LiOH, H_2_O, THF, 25 °C, 12 h.

In the design and synthesis of hapten, keeping the maximal extent of the parent structural characteristics is crucial to the ultimate acquisition of antibody with high sensitive and selectivity. Since FD, a novel molecule that targets ryanodine receptors with skeleton phthalamides, was developed as a commercial insecticide, many studies were conducted to evaluate the structure modification on the three moieties of FD’s, namely (a) phthalic acid, (b) aliphatic amine, and (c) aromatic amine. The previous study showed that the modification in phthalic acid and aromatic amine had negative influence on FD insecticidal activity, whereas modification in the aliphatic amine terminal could retain or improve FD insecticidal activity^19–21^. Based on these findings, carboxylic acid group was introduced to the terminal of aliphatic amine for binding to carrier protein, whereas phthalic acid and aromatic amine moieties were kept. With this method, we designed and synthesized the hapten (compound **10)**, as shown in Fig. 1. Firstly, compound **3** containing an amine group was prepared by the reaction of ethyl 3-mercaptopropanoate (compound **1**) with 2, 2-dimethylaziridine (compound **2**) in the 1, 2-dimethoxyethane, and then reacted with 3-iodophthalic anhydride (compound **4**) in the dichloromethane with triethylamine as the acid acceptor to obtain compound **5**. In the presence of trifluoroacetic anhydride as dehydrating agent, compound **5** was converted to corresponding N-substituted isoimide compound **6** at 0–5 °C, and subsequently reacted with 2-methyl-4-(perfluoropropan-2-yl)aniline (compound **7**) at 25 °C with a yield of 54% to give the compound **8**, which was oxidized by m-chloroperbenzoic acid (m-CPBA) to obtain compound **9**. Finally, compound **9** was hydrolyzed by aqueous lithium hydroxide to form compound **10** (FD-PA) containing a carboxylic acid group at yield of 85%.

**Figure 2:**
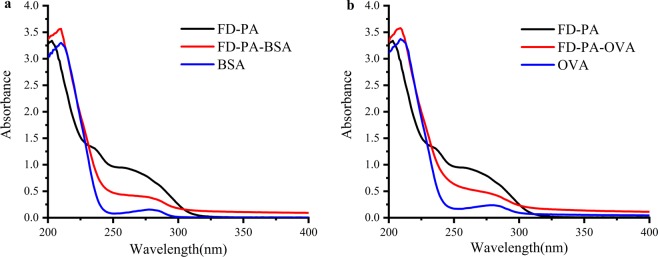
UV absorbance spectra of (**a**) FD-PA, FD-PA-BSA and BSA; (**b**) FD-PA, FD-PA-OVA and OVA.

We conjugated hapten (FD-PA) with BSA and OVA via the active ester method as immunogen and coating antigen, respectively. The UV-vis spectra were used to monitor the conjugation of haptens and carrier proteins. As shown in Fig. 2a, the hapten (FD-PA) showed main UV peaks at 203 nm, and BSA showed main UV peaks at 210 nm and 278 nm. The conjugate (FD-PA-BSA) showed peaks at 210 nm, but its shape of the UV absorption curve was different to that for the hapten (FD-PA) and BSA, which indicated successful conjugation. As same, Fig. 2b showed that the coating antigen FD-PA-OVA was obtained successfully, too. The molar ratios of FD hapten to proteins were estimated to be 12:1 and 10:1 for FD-PA-BSA and FD-PA-OVA, respectively.

Antisera collected from the mice after the fourth immunisation were screened against the coating antigen. The titre of the antibody was defined as the fold dilution with an absorbance of 1.0 in icELISA. The mouse with the highest titre and the best percentage inhibition in icELISA was used for further study. Three positive hybridoma cells were cloned by limiting dilution. A positive clone, designated as 4B3, was found to secret mAbs against artemisinin. Subsequently, 4B3 was cultivated and used to produce ascites.

### Optimization of icELISA conditions

To make icELISA work at optimal conditions, some influence parameters were optimized as below^22,23^. Regarding to the optimal combination of coating antigen and mAb, a checkerboard titration was performed in icELISA (see Supplementary Table S1), and the results showed that the dilution ratios of coating antigen (1 mg mL^−1^) and mAb 4B3 (1.0 mg mL^−1^) were at 1:500 and 1:2000, respectively. Thus, the concentration of FD-PA-OVA as the coating antigen was fixed as 2 µg ml^−1^ and the concentration of the mAb 4B3 was fixed at 0.5 µg ml^−1^ in this study.

**Figure 3:**
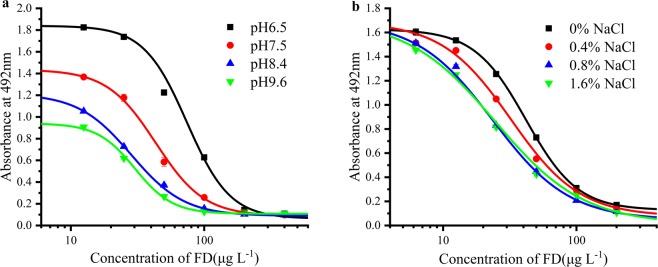
(**a**) Effect of pH on icELISA; (**b**) Effect of NaCl concentration on icELISA. Each point represents the mean of triplicate analyses. Vertical bars indicate mean ± standard deviations (SD).

The effect of pH values and NaCl concentrations on icELISA were studied, too. The maximum absorbance (A_max_), IC_50_, and A_max_/IC_50_ ratio were used to assess the optimum condition. As shown in Fig. 3a, four different pH values (6.5, 7.5, 8.4 and 9.6) were tested, and the A_max_/IC_50_ value at pH 8.4 reached the best with the lowest IC_50_ (28.4 µg L^−1^) compared to that at pH 6.5, 7.5 and 9.6, so pH 8.4 was selected as the optimal one. Regarding to the effect of ionic strength on established icELISA, as shown in Fig. 3b, we observed that the concentrations of NaCl (0, 0.4, 0.8 and 1.6 g L^−1^) had a little effect on A_max_ values. However, the IC_50_ decreased with increasing concentrations of NaCl from 0 to 0.8 g L^−1^, and when the NaCl concentration was increased to 1.6 g L^−1^ from 0.8 g L^−1^, the change of IC_50_ was very small. Therefore, 0.8% NaCl was chosen as the optimal ionic strength for the assay buffer.

**Figure 4:**
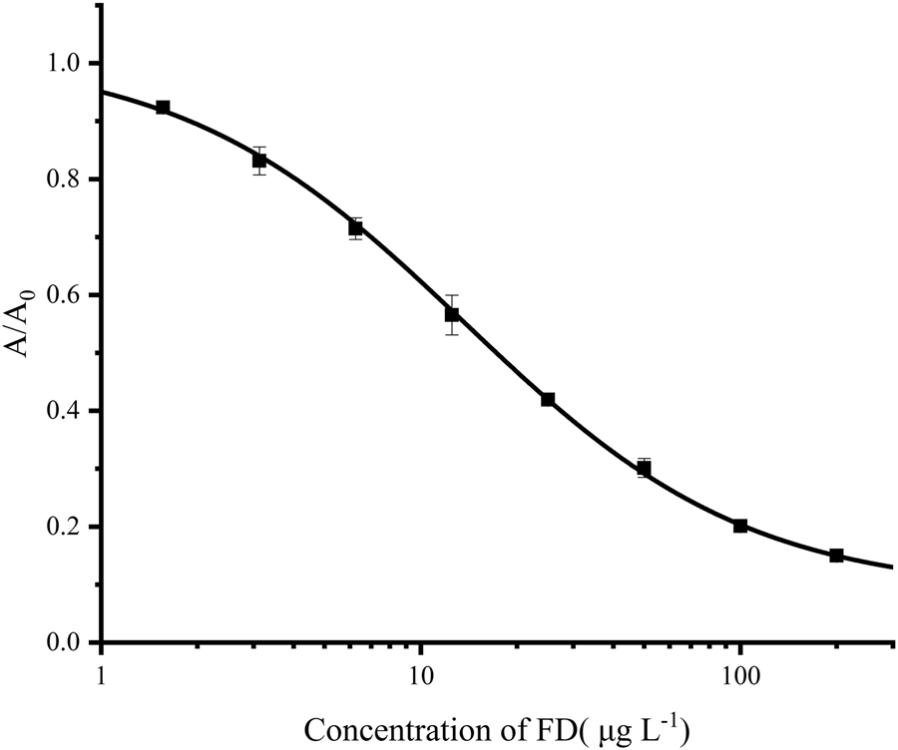
Standard inhibition curve of FD measured by using icELISA. A_0_ and A were absorbance in the absence and presence of competitors, respectively. Each point represents the mean of triplicate analyses. Vertical bars indicate mean ± standard deviations (SD).

Under the optimal conditions obtained above, a typical inhibition curve (see Fig. 4) was obtained with the IC_50_ value of 17.25 µg L^−1^, the detection limit of 1.89 µg L^−1^ (10% inhibition), and the working range between 4.06 and 103.59 µg L^−1^ (20% and 80% inhibition, respectively).

### Specificity of the mAb

To evaluate the specificity of icELISA for mAb 4B3, the cross-reactivity of icELISA with FD, chlorantraniliprole, cyantraniliprole, and other FD analogues were measured (see Supplementary Table S2). Although the three commercial insecticides used all target ryanodine receptors, the FD antibody specifically recognised FD, and showed no cross-reactivity with chlorantraniliprole and cyantraniliprole^24,25^. Meanwhile, the other FD analogues also showed no cross-reactivity with FD, indicating the validity of the novel haptens, and that the antibody was sufficiently specific for FD analysis.

### Recoveries of FD in spinach, tap water and soil samples

The recovery experiment of FD was conducted to determine the accuracy and reliability of icELISA. The average recoveries of FD by icELISA were 89.3–112.3%, 93.0–102.1%, and 86.9–97.6% in spinach, tap water, and soil, respectively (see Table 1). These findings showed that the established icELISA was a reliable, sensitive, and quick method of FD detection in food, tap water, and soil samples.

**Table 1:** Recoveries of FD in spinach, tap water and soil samples by the icELISA.

Samples	Spiked concentrations (ng mL^−1^ and ng g^−1^)	Detected concentrations (ng mL^−1^ and ng g^−1^)	Recovery (%) ± SD^a^
Spinach	1000	1123.0 ± 52.25	112.3 ± 5.2
	1500	1438.57 ± 16.47	95.9 ± 1.0
	2000	1927.37 ± 9.98	96.3 ± 0.5
	3000	2750.26 ± 34.72	91.6 ± 1.1
	4000	3573.4 ± 112.81	89.3 ± 2.8
Tap water	20	18.6 ± 0.63	93.0 ± 3.1
	30	28.61 ± 0.53	95.4 ± 1.7
	40	40.84 ± 0.82	102.1 ± 2.0
	60	56.16 ± 0.39	93.6 ± 0.6
	80	80.29 ± 1.2	100.3 ± 2.1
Soil	500	488.14 ± 18.14	97.6 ± 3.6
	1000	954.59 ± 31.16	95.4 ± 3.1
	1500	1362.85 ± 19.78	90.8 ± 1.3
	2000	1832.53 ± 47.63	91.6 ± 2.3
	3000	2608.27 ± 56.99	86.9 ± 1.8

### Comparison of icELISA and UPLC-MS/MS for analysis of FD in spinach and soil samples

**Figure 5:**
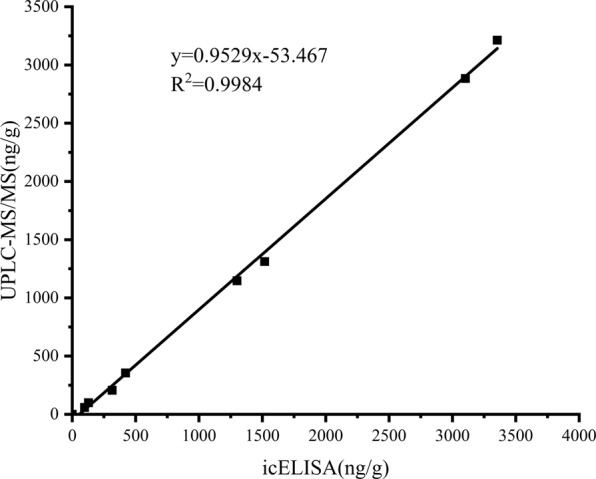
Correlation between icELISA and UPLC-MS/MS analysis for FD detection in spinach and soil samples.

By UPLC-MS/MS analysis, the retention time of FD was approximately 1.23 min and the m/z of 681.100 ([M-1]^−^) in the spectrogram confirmed the presence of FD (see Supplementary Fig. S1). Spinach and its soil sprayed with formulated FD product were collected to detect the residual FD by icELISA and UPLC-MS/MS (see Tables 2 and 3). The results showed that the residual FD at three days after spraying was higher in spinach than in the soil. The residual amount is significantly reduced due to prolonged time. The residual FD in outdoor samples were relatively slightly lower than that in indoor samples possibly due to environmental factors, such as sunlight and air flow. Spinach obtained from three different local supermarkets and soil collected from three provinces were tested with the same methods (see Tables 2 and 3). FD residue in these samples were not detectable (data not showed). Furthermore, the correlation coefficient (R^2^) between icELISA and UPLC-MS/MS was 0.9984 (see Fig. 5), showing that the contents detected by icELISA were in agreement with those detected by UPLC-MS/MS, suggesting that icELISA was reliable for the analysis of the residual FD.

**Table 2:** Determination of spinach samples by icELISA and UPLC-MS/MS.

Spinach^a^	icELISA(ng g^−1^)	UPLC-MS/MS(ng g^−1^)
1	3102.20 ± 44.58^b^	2883.5 ± 44.6
2	3353.57 ± 47.08	3213.2 ± 51.9
3	1300.71 ± 50.00	1146.22 ± 78.2
4	1518 ± 51.51	1311.59 ± 11.0
5–7	ND^c^	ND

^a^Each sample was analysed in triplicate. ^b^The data represented the mean ± SD. ^c^Not detected.

Sample 1–2: Spinach samples were collected outdoor and indoor on the third day after spraying.

Sample 3–4: Spinach samples were collected outdoor and indoor on the fifth day after spraying.

Sample 5–7: Spinach samples were bought from different local markets.

**Table 3:** Determination of soil samples by icELISA and UPLC-MS/MS

Soil^a^	icELISA(ng g^−1^)	UPLC-MS/MS(ng g^−1^)
1	314.89 ± 8.38^b^	206.32 ± 6.88
2	421.09 ± 11.88	354.66 ± 12.12
3	97.23 ± 0.99	58.25 ± 2.42
4	128.45 ± 1.93	98.88 ± 3.10
5–7	ND^c^	ND

^a^Each sample was analysed in triplicate. ^b^The data represented the mean ± SD. ^c^Not detected.

Sample 1–2: Soil samples were collected outdoor and indoor on the third day after spraying.

Sample 3–4: Soil samples were collected outdoor and indoor on the fifth day after spraying.

Sample 5–7: Soil samples were collected from different places.

## Conclusion

According to the European Food Safety Authority (EFSA), the maximum residue limit (MRL) of FD in spinach and the minimum MRL in foods or fruits are 15 µg g^−1^ and 10 ng g^−1^, respectively^26,27^. The detection limit of the icELISA method developed in this study reached 1.89 µg L^−1^ (equivalent to 1.89 ng g^−1^), which was 5-fold below the EPA minimum MRL. Thus, the sensitivity of the developed assay is sufficient for screening residual FD in practical applications and it could be used for quick and reliable detection of FD residues in environmental and agricultural samples.

In this study, FD haptens were designed and synthesised. Corresponding mAbs were also produced and used in icELISA development for FD detection. The established assay exhibited no cross-reactivity with insect ryanodine receptors-targeting chlorantraniliprole and cyantraniliprole, and showed no cross-reactivity with several FD analogues. Developed based on mAb 4B3, the icELISA method had an IC_50_ value of 17.25 µg L^−1^ for FD and a working range of 4.06–103.59 µg L^−1^ based on 20–80% of inhibition. In addition, the fortified FD detected by icELISA in spinach, tap water, and soil samples had average recoveries of 86.9–112.3%. Moreover, the amount of residual FD in actual spinach and soil samples detected by icELISA was in agreement with that detected by UPLC-MS/MS (R^2^ > 0.99). In conclusion, the results displayed that this new immunoassay was suitable for the quick and convenient determination of residual FD in environmental and agricultural samples.

## Materials and Methods

### Reagents and apparatus

FD was purchased from Nichino Shanghai Co., Ltd. FD analogues were synthesised in our previous work and characterised by using ^1^H NMR, ^13^C NMR and HRMS. FD suspension concentrate (SC, 20%) was purchased from the China National Agricultural Means of Production Group Corporation. Chlorantraniliprole and cyantraniliprole were purchased from Shanghai DuPont (China). Reagents purchased from Sigma-Aldrich (St. Louis, MO) included the serum-free cell-freezing medium dimethylsulphoxide (DMSO), bovine serum albumin (BSA), ovalbumin (OVA), hypoxanthine, aminopterin, thymidine (HAT), penicillin, streptomycin, L-glutamine, complete and incomplete Freund’s adjuvant, polyoxyethylene sorbitan monolaurate (Tween-20), and N-hydroxysuccinimide (NHS). Polyethyleneglycol (PEG)-2000, o-phenylenediamine (OPD), N, N-dicyclohexylcarbodiimide (DCC), 3-iodophthalic anhydride, ethyl 3-mercaptopropanoate, 2,2-dimethylaziridine, 2-methyl-4-(perfluoropropan-2-yl)aniline and m-chloroperbenzoic acid (m-CPBA) were purchased from J & K Chemical (Beijing, China). Goat anti-mouse IgG conjugated with horseradish peroxidase (IgG–HRP) was purchased from Jackson Immuno Research Laboratories (West Grove, PA). Dulbecco’s modified Eagle’s medium (DMEM) and fetal bovine serum (FBS) were obtained from Gibco BRL (Paisley, Scotland). Chromatography-grade acetonitrile was purchased from Thermo Fisher Scientific (New Jersey, USA). All other reagents were obtained from Beijing Chemical Reagents Co. (Beijing, China).

Cell culture plates and 96-well polystyrene microplates were purchased from Costar (Corning, NY). The direct heat CO_2_ incubator (Thermo, 311), automated plate washer (Wellwash 4 Mk2), and microplate reader (Multiskan MK3) were purchased from Thermo Fisher Scientific (Vantaa, Finland). A KH-500E ultrasonic cleaner was purchased from Kunshan Hechuang Ultrasonic Apparatus Co., Ltd. (Jiangsu, China). The electric heating constant temperature incubator was purchased from Shanghai Zhicheng Analytical Instrument Manufacturing Co., Ltd. (Shanghai, China).

### Immunoreaction buffers and solutions

The buffers and solutions included coating buffer (0.05 M carbonate buffer, pH 9.6), phosphate-buffered saline (PBS) (0.1 M phosphate buffer containing 0.9% NaCl, pH 7.5), PBS with 0.1% (v/v) Tween-20 (PBST), PBST containing 0.5% (w/v) gelatin (PBSTG), citrate-phosphate buffer (0.01 Mcitric acid and 0.03 M Na_2_HPO_4_, pH 5.5), substrate solution (4 mL 30% H_2_O_2_ added to 10 mL citrate-phosphate buffer containing 2 mg mL^−1^ OPD), and stop solution (2.0 M H_2_SO_4_). FD standard solutions of various concentrations were prepared by diluting the 1 mg mL^−1^ stock solution (FD powder dissolved in DMSO) with methanol. All reagents and solvents were of analytical grade. Deionised water used for diluting buffers and solutions was produced by using Millipore Water Purification System (Millipore Co., Billerica, MA).

### Myeloma cell line and cell culture mediums

For the fusion experiments, SP2/0-Ag14 cells (HAT sensitive mouse myeloma cell line) were obtained from the China Institute of Veterinary Drug Control (Beijing, China). For cultivation of myeloma and hybridoma cells, DMEM containing 10–20% (v/v) FBS was supplemented with 0.2 M glutamine, 50 000 units L^−1^ penicillin, and 50 mg L^−1^ streptomycin.

### Synthesis of FD hapten

Hapten was synthesised^19,28^ according to the synthetic route illustrated in Fig. 1.

#### Synthesis of ethyl 3-((2-amino-2-methylpropyl) thio)propanoate (compound **3**)

Compound **1** (6.7 g, 50 mmol) and compound **2** (3.55 g, 50 mmol) were added into 100 mL 1, 2-dimethoxyethane in a 250 mL flask. The mixture was heated at 85 °C and stirred for 3 h. The solvent was vacuum-evaporated, whereas the residue was then purified by silica gel column chromatography (methanol-dichloromethane, 1:20) to obtain compound **3** in light yellow liquid form (8.8 g, 86%). ^1^H NMR (300 MHz, Chloroform-d): δ 4.07 (q, *J* = 7.1 Hz, 2H), 2.75 (t, *J* = 7.6 Hz, 2H), 2.56–2.48 (m, 4H), 1.44 (s, 2H), 1.19 (t, *J* = 7.1 Hz, 3H), 1.09 (s, 6H).

#### Synthesis of 2-((1-(2-ethoxycarbonylethylthio)-2-methylpropan-2-yl)carbamoyl)-3-iodobenzoic acid (compound **5**)

A mixture of compound **3** (5.12 g, 25 mmol) and triethylamine (2.53 g, 25 mmol) in dichloromethane (80 mL) was slowly added to a compound **4** solution (6.85 g, 25 mmol) in dichloromethane (80 mL) at 25 °C. The reaction mixture was stirred for 5 h, poured into water (100 mL), and acidified with diluted hydrochloric acid (2 M, 20 mL) to pH 2–3. The aqueous layer was extracted three times with dichloromethane (30 mL) and dried over anhydrous sodium sulphate. The solvent was vacuum-evaporated and the residue was used in the subsequent reaction without further purification.

#### Synthesis of ethyl 3-(2-((7-iodo-3-oxoisobenzofuran-1(3H)-ylidene)amino)-2-methylpropylthio)propanoate (compound **6**)

Slurry of compound **5** residue in 60 mL anhydrous toluene was cooled with an ice bath. Trifluoroacetic anhydride (5.25 g, 25 mmol) was added dropwise to the mixture with stirring. Following completion of the addition, the mixture was further stirred for 2 h. The progress of the reaction was monitored by using TLC (petroleum ether/ethyl acetate = 1:1). After the reaction was complete, the solvent was vacuum-evaporated, whereas the residue was used in the subsequent reaction without further purification.

#### Synthesis of ethyl 3-(2-(2-iodo-6-((2-methyl-4-(perfluoropropan-2-yl)phenyl)carbamoyl)benzamido)-2-methylpropylthio)propanoate (compound **8**)

Compound **7** (6.85 g, 25 mmol) was added to compound **6** residue solution in dichloromethane (30 mL). Two drops of trifluoroacetic acid was added to the mixture, which was subsequently stirred for 1 h at 25 °C. The progress of the reaction was monitored by using TLC (petroleum ether/ethyl acetate, 2:1). Following completion of the reaction, the solution was washed with diluted hydrochloric acid (2 M, 20 mL), aqueous sodium carbonate solution (2 M, 20 mL), and water (30 mL), respectively, and dried over anhydrous sodium sulphate. The solvent was vacuum-evaporated, whereas the residue was purified by silica gel column chromatography (petroleum ether/ethyl acetate, 2:1) to obtain compound **8** in white solid form (9.9 g, 54%). ^1^H NMR (300 MHz, DMSO-*d*_6_): δ 9.57 (s, 1H), 8.15 (s, 1H), 8.03 (s, 1H), 8.01–7.98 (m, 1H), 7.68 (d, *J* = 8.2 Hz, 1H), 7.53 (s, 2H), 7.26 (t, *J* = 7.8 Hz, 1H), 4.06 (q, *J* = 7.1 Hz, 2H), 2.93 (s, 2H), 2.62 (t, *J* = 7.2 Hz, 2H), 2.47 (t, *J* = 7.2 Hz, 2H), 2.38 (s, 3H), 1.32 (s, 6H), 1.18 (t, *J* = 7.1 Hz, 3H). ^13^C NMR (75 MHz, DMSO-*d*_6_): δ 171.30, 167.67, 165.75, 141.59, 140.79, 139.63, 136.14, 131.98, 130.12, 127.49, 127.32, 127.18, 124.01, 123.50, 123.36, 121.30, 121.02, 95.21, 89.93, 89.50, 60.06, 54.72, 42.05, 34.56, 28.63, 25.44, 18.03, 14.08. HRMS (m/z): [M-H]^−^ calcd. for C_27_H_28_F_7_IN_2_O_4_S, 735.0630; found, 735.0633.

#### Synthesis of ethyl 3-((2-(2-iodo-6-((2-methyl-4-(perfluoropropan-2-yl)phenyl)carbamoyl)benzamido)-2-methylpropyl)sulphonyl)propanoate (compound **9**)

M-Chloroperoxybenzoic acid (3.6 g, 21 mmol) was added to compound **8** solution (7.36 g, 10 mmol) in dichloromethane (60 mL). The reaction was carried out at 25 °C and the progress of the reaction was monitored by using TLC (petroleum ether/ethyl acetate = 1: 2). Following completion of the reaction, the mixture was poured into water and extracted with chloroform. The organic layer was washed with an aqueous sodium hydrosulphite solution (2 M, 30 mL) and an aqueous sodium carbonate solution (2 M, 30 mL), respectively, and dried over anhydrous sodium sulphate. The solvent was vacuum-evaporated, whereas the residue was purified by silica gel column chromatography (petroleum ether/ethyl acetate, 1:2) to obtain compound **9** in white solid form (7 g, 92%). ^1^H NMR (300 MHz, DMSO-*d*_6_): δ 9.78 (s, 1H), 8.44 (s, 1H), 8.03 (dd, *J* = 7.9, 0.9 Hz, 1H), 7.89 (d, *J* = 8.3 Hz, 1H), 7.74 (d, *J* = 7.2 Hz, 1H), 7.53 (d, *J* = 8.0 Hz, 2H), 7.29 (t, *J* = 7.8 Hz, 1H), 4.10 (q, *J* = 7.1 Hz, 2H), 3.70 (s, 2H), 3.39–3.34 (m, 2H), 2.68 (t, *J* = 7.3 Hz, 2H), 2.39 (s, 3H), 1.54 (s, 6H), 1.19 (t, *J* = 7.1 Hz, 3H). ^13^C NMR (75 MHz, DMSO-*d*_6_): δ 170.16, 167.81, 165.71, 141.50, 140.90, 139.52, 136.09, 133.00, 130.26, 127.42, 127.25, 125.00, 123.49, 123.36, 121.71, 121.44, 120.43 (dd, *J* = 286.9, 28.0 Hz), 95.45, 91.26 (dt, *J* = 200.7, 32.5 Hz), 79.28, 60.68, 59.03, 52.66, 50.46, 26.72, 26.31, 18.09, 14.01. HRMS (m/z): [M-H]^−^ calcd. for C_27_H_28_F_7_IN_2_O_6_S, 767.0528; found, 767.0533.

#### Synthesis of 3-((2-(2-iodo-6-((2-methyl-4-(perfluoropropan-2-yl)phenyl)carbamoyl)benzamido)-2-methylpropyl)sulphonyl)propanoic acid (flubendiamide hapten) (compound **10**)

LiOH (2 M, 15 mL) was added dropwise to compound **9** solution (3.84 g, 5 mmol) in THF (40 mL) at 0 °C. The reaction mixture was stirred for 12 h. The solvent was vacuum-evaporated and the residue was acidified with diluted hydrochloric acid (2 M, 20 mL). The aqueous layer was extracted three times with dichloromethane (20 mL) and dried over anhydrous sodium sulphate. The solvent was vacuum-evaporated and the residue was purified by silica gel column chromatography (ethyl acetate: methanol = 20:1) to obtain compound **10** in white solid form (3.12 g, 85%). ^1^H NMR (300 MHz, DMSO-*d*_6_): δ 9.79 (s, 1H), 8.43 (s, 1H), 8.00 (dd, *J* = 7.9, 0.9 Hz, 1H), 7.85 (d, *J* = 9.2 Hz, 1H), 7.71 (d, *J* = 7.6 Hz, 1H), 7.51 (d, *J* = 7.7 Hz, 2H), 7.26 (t, *J* = 7.8 Hz, 1H), 3.67 (s, 2H), 3.30 (t, *J* = 7.4 Hz, 2H), 2.59 (t, *J* = 7.4 Hz, 2H), 2.36 (s, 3H), 1.51 (s, 6H). ^13^C NMR (75 MHz, DMSO-*d*_6_): δ 171.65, 167.78, 165.71, 141.49, 140.87, 139.51, 136.07, 132.99, 130.26, 127.41, 127.27, 124.99, 123.53, 123.40, 121.70, 95.47, 79.30, 58.97, 52.67, 50.73, 26.82, 26.29, 18.10. HRMS (m/z): [M-H]^−^ calcd. for C_25_H_24_F_7_IN_2_O_6_S, 739.0215; found, 739.0212.

### Synthesis of FD-protein conjugates

Through the carbodiimide method, hapten was conjugated with BSA and OVA to produce immunogen (FD–BSA) and coating antigen (FD–OVA)^29^. The mixture was dialysed against 3 L PBS for 3 days with three solvent changes per day, and stored at -20 °C. The conjugates of FD−OVA and FD−BSA were used as a coating antigen and immunogen, respectively. By using UV-vis spectroscopy, the final structures of the protein-conjugated haptens were confirmed^30^.

### Immunisation protocol, monoclonal antibody production, and purification

A mAb against artemisinin was prepared according to the procedures described previously by Zhao in 2011^31^. Five female Balb/c mice aged seven weeks old were immunised with subcutaneous injection of 100 µg FD-OVA dissolved in 0.1 mL PBS and 0.1 mL complete Freund’s adjuvant. Incomplete Freund’s adjuvant was used in the two subsequent FD-OVA injections at 2-week intervals. Two weeks after the third injection, the mouse with the best immune performance was injected with 100 µg FD-BSA in 100 µL PBS. Three days later, spleen cells were collected from the mouse, mixed with the SP2/0-Ag14 myeloma cells at a ratio of 10:1, and fused with PEG-2000. Next, the cells were cultured in complete medium (DMEM supplemented with 15% FBS, 0.2 M glutamine, 50 000 units L^−1^ penicillin, and 50 mg L^−1^ streptomycin). The hybridoma cells were selectively cultured in complete medium supplemented with HAT for 7 days. Next, the supernatant was subjected to icELISA, in which positive hybridoma cells were cloned by limiting dilution. The clones were further selected by icELISA. The clone with high antibody titre and good sensitivity in the culture supernatant was designated as 4B3 and cultured in mice for production of mAb in ascites. Anti-FD mAbs were purified from ascite fluid by ammonium sulphate precipitation.

### Indirect competitive ELISA

A checkerboard titration procedure was used to optimize coating antigen and anti-FD mAb interaction. All plates were washed four times with washing buffer (PBST) and all reactions were incubated in a constant-temperature incubator at 37 °C. First, the microplate was coated with FD–PA-OVA (100 µL) in coating buffer at 37 °C for 3 h. After the microplate was washed, standard sample or analytes in PBSTG (50 µL) was added to each microplate well, followed by addition of mAb in PBSTG (50 µL). Following incubation for 0.5 h, the microplate was washed with PBST. Each well was incubated with goat anti-mouse IgG–HRP (1 mg/L) diluted in PBSTG (100 µL) at a ratio of 1: 1 000 for 0.5 h. Next, the plate was washed again and substrate solution (100 µL) was added to each well. Finally, 15 min later, the reaction was terminated by adding stop solution (50 µL, 2.0 M sulphuric acid) to each well, and the absorbance was measured by using a microplate reader at 492 nm.

Experimental parameters, including buffer pH and ionic strength, were studied to improve the sensitivity of the immunoassay. The influences of pH values were evaluated using different PBSTG solutions with pH values (6.5, 7.5, 8.4, and 9.6). The effects of ionic strength were determined using PBSTG buffer at different NaCl concentration (0, 0.4%, 0.8% and 1.6% by mass fraction). Standard curves were obtained using Origin 2018 software.

### Antibody specificity against FD

The mAb specificity of icELISA was evaluated by cross-reactivity (CR) with a set of FD structural analogues. The relative CR was calculated by the following formula:

$${\rm{CR}}=\frac{{{\rm{IC}}}_{{\rm{50}}}({\rm{FD}})}{{{\rm{IC}}}_{{\rm{50}}}({\rm{analogue}}\,{\rm{of}}\,{\rm{FD}})}\times \mathrm{100} \% $$

### Preparation of spinach and soil samples

FD (SC, 20%) was sprayed according to label directions at dosage of 250 g a.i.ha^−1^ on the spinach cultivated in a greenhouse and an outdoor experimental field of China Agricultural University. On the third and fifth days after spraying, spinach samples and soil samples were collected. Meanwhile, three spinach samples were purchased from different supermarkets and three soil samples were separately collected from Xiaotangshan (Beijing), Wuqiao (Hebei), and Jiaozuo (Henan). All samples were stored at −40 °C until use.

### Sample preparation and extraction

Chopped and homogenised spinach leaves (1 g) and grinded soil (1 g) were placed into 10 mL polytetrafluoroethylene centrifuge tubes. Following addition of 3 mL acetonitrile, the mixture was allowed to stand at 4 °C for 3 h. Next, the mixture was stirred on a vortex shaker for 3 min, monitored by ultrasonic extraction for 10 min, and centrifuged at 4 000 × *g* for 5 min. The organic layer was dehydrated by filtration through anhydrous sodium sulphate. The same extraction steps were repeated three times. All extracts were combined and dried by a gentle stream of nitrogen gas. The residues were dissolved in PBSTG (1.0 mL) and were diluted 90- or 45-folds for use in icELISA. Each analysis was conducted in triplicate.

### Recovery tests for FD spiked in tap water, soil, and spinach samples

Spinach leaves (1 g) was cut into pieces and then spiked with FD at concentrations of 1 000, 1 500, 2 000, 3 000 and 4 000 ng g^−1^. Tap water was spiked at concentrations of 20, 30, 40, 60, 80 ng mL^−1^. The grinded soil (1 g) was spiked at concentrations of 500, 1 000, 1 500, 2 000 and 3 000 ng g^−1^. FD in spiked samples were analysed with the established icELISA.

### UPLC-MS/MS analysis

FD standards, as well as spinach and soil samples were analysed with UPLC-MS/MS according to the procedure described by Caboni with slight modification^32^. The analysis was carried out by a Thermo Fisher TSQ Quantum Access MAX system (Tewksbury, Massachusetts, USA). Chromatographic separation was performed by a DionexTM UltiMateTM 3000 Open Sampler XRS UHPLC system with a Hypersil GOLD C18 column (100 mm × 2.1 mm i.d., 3 µm particle size). The mobile phase was acetonitrile/water (90:10, v/v) at a flow rate of 0.3 mL min^−1^. The injection volume was 5 µL. A triple quadrupole mass spectrometer with an electrospray ionisation source was applied for analysis under the following conditions: spray voltage of 3000 V, capillary temperature of 350 °C, vaporiser temperature of 200 °C, sheath gas pressure of 35 psi, and aux valve flow of 15 arb. The collision gas was Argon (99.999%) with pressure at 1.5 mTorr. All analytes were ionized in negative mode. The UPLC-MS/MS results were compared with those of icELISA to evaluate the reliability of the established immunoassay.

### Ethical approval

All procedures performed in studies involving animals were in strict accordance to the standards described in the “Guide for the Care and Use of Laboratory Animals” (National Research Council Commission on Life Sciences, 1996 edition). Animal studies were approved by the independent Animal Ethical Committee at China Agricultural University.

## Supplementary information

Supplementary information

## Data Availability

All data generated or analysed during this study are included in this published article (and its Supplementary Information files).
