# Supplementary material for: A monoclonal antibody-based indirect competitive enzyme-linked immunosorbent assay for flubendiamide detection
Source: Sci Rep. 2019 Feb 14;9:2131. doi: 10.1038/s41598-019-38649-w (PMC6376021; doi:10.1038/s41598-019-38649-w)
Supplement: Supplementary file 1 — Supplementary information [file 41598_2019_38649_MOESM1_ESM.docx]

**A monoclonal antibody-based indirect competitive enzyme-linked immunosorbent assay for flubendiamide detection**

Qibo Li ^1^, Yongliang Cui^2^, Min Liao^1^, Tong Feng^1^, Guiyu Tan^3^, Baomin Wang^3^, Shangzhong Liu^1*^

^1^*College of Science, China Agricultural University, Beijing 100193, China*

^2^*Citrus Research Institute, Southwest University, Chongqing 400712, China*

^3^*College of Agronomy and Biotechnology, China Agricultural University, Beijing 100193, China*

^*^Corresponding author. E-mail: [shangzho@cau.edu.cn](mailto:shangzho@cau.edu.cn). Tel: +86-10-62731070. Fax: +86-10-62731070

**Contents**

[Table S1. Checkerboard titration of coating antigen and mAb with different dilution ratios. 2](#_Toc528259078)

[Table S2. Assay cross-reactivity of FD and its analogues. 2](#_Toc528259079)

[Fig. S1. Selected ion monitor scan spectra and secondary ion mass spectrometry of FD on UPLC-MS/MS 4](#_Toc528259080)

[Characterization of compound A_1_, A_2_, A_3_ and A_4_ 5](#_Toc528259081)

[^1^H and ^13^C NMR Spectra of All Compounds 5](#_Toc528259082)

[HRMS Spectra of All Compounds 13](#_Toc528259083)

# Table S1. Checkerboard titration of coating antigen and mAb with different dilution ratios.

| Dilution ratios of coating antigen | Dilution ratios of mAb | | | | | | | | | |
| --- | --- | --- | --- | --- | --- | --- | --- | --- | --- | --- |
|  | 1:500 | | 1:1000 | | 1:2000 | | 1:4000 | | 1:8000 | |
|  | C | I | C | I | C | I | C | I | C | I |
| 1:500 | 2.206 | 0.923 | 1.651 | 0.522 | 1.041 | 0.324 | 0.439 | 0.209 | 0.215 | 0.143 |
| 1:1000 | 2.060 | 0.517 | 1.255 | 0.251 | 0.711 | 0.170 | 0.488 | 0.144 | 0.265 | 0.085 |
| 1:2000 | 1.790 | 0.335 | 0.955 | 0.185 | 0.518 | 0.122 | 0.398 | 0.101 | 0.185 | 0.070 |
| 1:4000 | 1.380 | 0.210 | 0.768 | 0.124 | 0.406 | 0.092 | 0.305 | 0.075 | 0.171 | 0.064 |
| 1:8000 | 0.965 | 0.139 | 0.575 | 0.094 | 0.312 | 0.072 | 0.228 | 0.067 | 0.135 | 0.056 |

C: absorbance of negative well. I: absorbance of FD well.

# Table S2. Assay cross-reactivity of FD and its analogues.

| Chemical names | Structures | IC_50_(µg L^-1^) | CR (%) |
| --- | --- | --- | --- |
| 3-iodo-N^2^-(2-methyl-1-(methylsulphonyl)propan-2-yl)-N^1^-(2-methyl-4-(perfluoropropan-2-yl)phenyl)phthalamide  (flubendiamide) |  | 17.25 | 100 |
| 3-bromo-N-(4-chloro-2-methyl-6-(methylcarbamoyl)phenyl)-1-(3-chloropyridin-2-yl)-1H-pyrazole-5-carboxamide  (chlorantraniliprole) |  | NI | NI |
| 3-bromo-1-(3-chloropyridin-2-yl)-N-(4-cyano-2-methyl-6-(methylcarbamoyl)phenyl)-1H-pyrazole-5-carboxamide  (cyantraniliprole) |  | NI | NI |
| 4,5-dichloro-N^1^-(2,6-dichlorophenyl)-N^2^-(2-methyl-1-(methylthio)propan-2-yl)phthalamide  (**A_1_**) |  | NI | NI |
| 4,5-dichloro-N^1^-(2,6-dichlorophenyl)-N^2^-(2-methyl-1-(methylsulphonyl)propan-2-yl)phthalamide  (**A_2_**) |  | NI | NI |
| 4,5-dichloro-N^1^-(2,6-dichlorophenyl)-N^2^-(2-methyl-1-(prop-2-yn-1-ylthio)propan-2-yl)phthalamide |  | NI | NI |
| (**A_3_**)  N^1^-(5-chloro-2-nitrophenyl)-3-iodo-N^2^-(2-methyl-1-(methylthio)propan-2-yl)phthalamide  (**A_4_**) |  | NI | NI |

NI: no inhibition was observed at up to 10,000 µg L^-1^of the compound

# Fig. S1. Selected ion monitor scan spectra and secondary ion mass spectrometry of FD on UPLC-MS/MS

# Characterization of compound A_1_, A_2_, A_3_ and A_4_

Compound **A_1_**: ^1^H NMR (300 MHz, DMSO-*d*_6_): δ 10.48 (s, 1H), 8.18 (s, 1H), 7.95 (s, 1H), 7.72 (s, 1H), 7.58 (d, *J* = 7.9 Hz, 2H), 7.40 (dd, *J* = 8.6, 7.6 Hz, 1H), 3.00 (s, 2H), 2.11 (s, 3H), 1.35 (s, 6H). ^13^C NMR (75 MHz, DMSO-*d*_6_): δ 165.51, 164.03, 138.34, 134.38, 133.92, 132.99, 132.60, 131.60, 130.37, 130.12, 129.54, 128.69, 54.81, 43.92, 26.13, 17.22. HRMS (m/z): [M-H]^-^ calcd. for C_19_H_18_Cl_4_N_2_O_2_S, 476.9770; found, 476.9778

Compound **A_2_:** ^1^H NMR (300 MHz, DMSO-*d*_6_): δ 10.49 (s, 1H), 8.43 (s, 1H), 7.95 (s, 1H), 7.82 (s, 1H), 7.58 (d, *J* = 8.0 Hz, 2H), 7.44 – 7.36 (m, 1H), 3.76 (s, 2H), 3.00 (s, 3H), 1.49 (s, 6H).

^13^C NMR (75 MHz, DMSO-*d*_6_): δ 165.75, 163.93, 138.21, 134.39, 133.94, 133.00, 132.60, 131.60, 130.57, 129.99, 129.58, 128.72, 59.32, 52.23, 43.28, 27.17. HRMS (m/z): [M-H]^-^ calcd. for C_19_H_18_Cl_4_N_2_O_4_S, 508.9669; found, 508.9675

Compound **A_3_:** ^1^H NMR (300 MHz, DMSO-*d*_6_): δ 10.48 (s, 1H), 8.19 (s, 1H), 7.95 (s, 1H), 7.71 (s, 1H), 7.58 (d, *J* = 7.9 Hz, 2H), 7.40 (dd, *J* = 8.7, 7.5 Hz, 1H), 3.37 (d, *J* = 2.6 Hz, 2H), 3.19 – 3.07 (m, 3H), 1.35 (s, 6H). ^13^C NMR (75 MHz, DMSO-*d*_6_): δ 165.57, 163.96, 138.37, 134.34, 133.94, 132.99, 132.60, 131.57, 130.38, 130.09, 129.54, 128.70, 80.94, 79.28, 73.61, 54.32, 41.33, 26.21, 20.23. HRMS (m/z): [M-H]^-^ calcd. for C_21_H_18_Cl_4_N_2_O_2_S, 500.9770; found, 500.9781

Compound **A_4_:** ^1^H NMR (300 MHz, DMSO-*d*_6_): δ 10.59 (s, 1H), 8.18 – 8.10 (m, 3H), 8.05 (dd, *J* = 7.9, 1.0 Hz, 1H), 7.69 (dd, *J* = 7.7, 1.0 Hz, 1H), 7.48 (dd, *J* = 8.9, 2.3 Hz, 1H), 7.30 (t, *J* = 7.8 Hz, 1H), 2.88 (s, 2H), 2.02 (s, 3H), 1.31 (s, 6H). ^13^C NMR (75 MHz, DMSO-*d*_6_): δ 167.79, 167.30, 146.79, 145.35, 140.34, 135.42, 133.35, 131.20, 129.43, 127.63, 122.62, 117.76, 115.64, 89.52, 61.17, 44.39, 26.61, 16.96. HRMS (m/z): [M+H]^+^ calcd. for C_19_H_19_ClIN_3_O_4_S, 547.9902; found, 547.9894

# ^1^H and ^13^C NMR Spectra of All Compounds

Compound **3**

Compound **8**

Compound **9**

Compound **10**

Compound **A_1_**

Compound **A_2_**

Compound **A_3_**

Compound **A_4_**

# HRMS Spectra of All Compounds

Compound **8**

Compound **9**

Compound **10**

Compound **A_1_**

Compound **A_2_**

Compound **A_3_**

Compound **A_4_**
